# Malaysian Journal of Medical Sciences’ Performance Status in 2018

**DOI:** 10.21315/mjms2019.26.4.1

**Published:** 2019-08-29

**Authors:** Nour Azimah Zulkapli, Jafri Malin Abdullah

**Affiliations:** 1 Malaysian Journal of Medical Sciences, Universiti Sains Malaysia, Pulau Pinang, Malaysia; 2 Malaysian Journal of Medical Sciences, Universiti Sains Malaysia, Kubang Kerian, Kelantan, Malaysia

**Keywords:** medical sciences, information, report, statistics

## Abstract

The Malaysian Journal of Medical Sciences (MJMS) would like to present a brief report of its progress in 2018 with a purpose to provide a clear picture of how it has performed so far. This report may considered as a helpful information, especially, to future authors who wish to submit their articles to MJMS. This report summarised the information of the total of original manuscripts received based on manuscript type, authors’ country of origin and total of original manuscripts received by month. It also reveals the statistics of the final decisions made based on manuscript type, the accept-reject ratio by the editor and the time taken from submission to decision for all manuscripts submitted throughout 2018.

## Introduction

All this while, the Editorial Office (EO) of Malaysian Journal of Medical Sciences (MJMS) received many enquiries, especially from new authors, regarding the SCImago Journal Rank (SJR) indicator and the quartile score of MJMS. Although these information could be easily found via the internet, it seemed that they are assuming that EO would have known better and able to provide the required information in just a second.

SJR indicator is a measure of journal’s impact, influence or prestige. It expresses the average number of weighted citations received in the selected year by the documents published in the journal in the three previous years. The SJR of MJMS in 2018 is Q3 with a slight decrease in score which is 0.273, compared to Q3 with 0.295 score in 2017 and Q3 with 0.285 score in 2016 (1).

Whereas, the H-index is the journal’s number of articles (h) that have received at least h citations over the whole period. As in the case of MJMS, it obtained H-index of 19 which considered all type of manuscripts published in 2018. This index score, indeed, has been maintained for quite a number of years (1).

Lately, MJMS continues to receive an overwhelmed response with an increase of total of manuscript submission despite the decrease of Q3 score in 2018. This, again, proves that MJMS is still recognised by medical peer groups in other parts of the world and it has been considered as the most reliable medium for dissemination of their research work.

## Submission Pattern by Month

**Figure 1:**
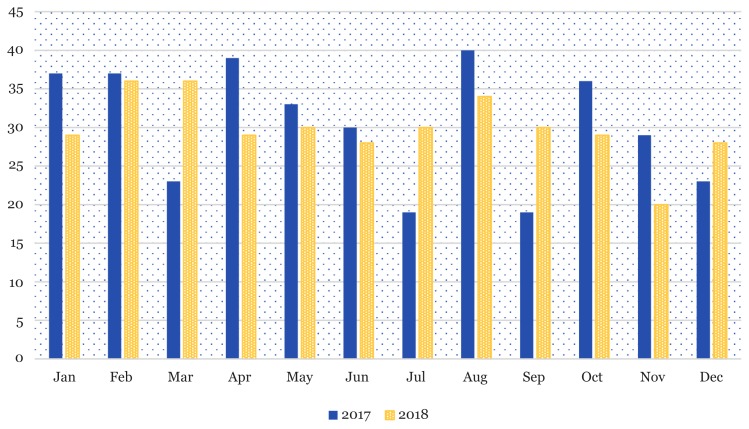
Original manuscripts submitted via ScholarOne Manuscripts™ system in 2017 and 2018. Source: https://mc.manuscriptcentral.com/maljms

Figure 1 shows that February and March 2018 were the months with highest total manuscript submissions, which is 36, respectively, followed by August with 34 manuscript submissions. Whereas, in year 2017, August received the highest manuscript submissions which was 40 followed by April with 39 manuscript submissions and, January and February with 37 manuscript submissions, respectively (2).

Although the total of manuscript submissions generated by the system in year 2018 shows a slight decrease which was 359 compared to year 2017 with 365 manuscripts but the actual total submission was 389 which included withdrawal, unsubmission and early rejection of manuscripts after preliminary screening done by the EO.

## Submission Pattern by Manuscript Type

As shown in Table 1, the Original Articles are the type of manuscript most submitted to MJMS with total of submission keep on increasing every year. In year 2017, MJMS received a total of 273 Original Articles and in 2018, the total increased to 290 manuscripts. Again, Review Articles are the second most submitted type of manuscript to MJMS although there is a slight decrease in 2018 compared to year 2017. MJMS rarely received Editorial manuscript type via the submission system and it is hard to obtain one from authors in order to have a complete issue for publication. The actual number of Editorial manuscripts received each year was 6 since it has to be published in every six issues and most of them were received through email.

## Submission Pattern by Country

MJMS was known as one of the established publication platform in Malaysia to researchers in the circle of Medical Sciences, for them to disseminate knowledge based on their new and extended research. In regard to this, most of the submitted manuscripts, which are 142 (Table 2), were from the Malaysian authors. Authors from other countries, such as Islamic Republic of Iran, India Indonesia, Nigeria, Saudi Arabia still remained the main contributors of MJMS.

## Time from Submission to Decision

There was time when EO received many queries regarding the time span of decision making of an article from day one of submission until a decision is made by the Editor-in-Chief.

**Figure 2:**
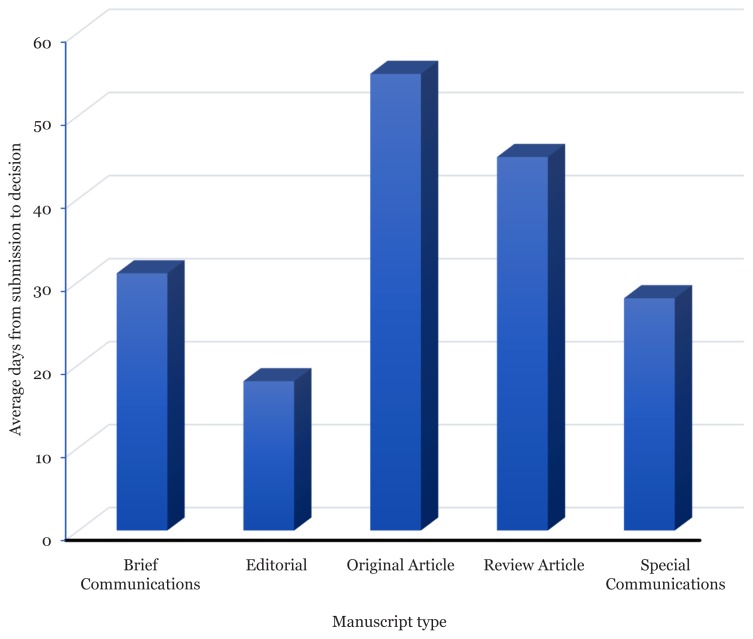
Average days from submission to decision by all type of manuscripts submitted between 1 Jan 2018 and 31 Dec 2018 Source: https://mc.manuscriptcentral.com/maljms

As shown in Figure 2, Original Articles take approximately 55 days to undergo reviewing process before a decision is made based on the collective reviewers’ comments. In most cases, reviewed manuscripts need be revised, although in the first place, they were thought to be perfectly written. The circle of reviewing process keeps on moving until all reviewers are satisfied and acknowledged the refinement of content has been done as suggested. Considering this, it is not impossible to have manuscripts still remained in several revision stages for so many years before the content of the manuscripts are solid enough and qualified to be published. This is what MJMS has been practice in order to maintain the quality of this journal.

## The Acceptance-Rejection Ratio

The statistics generated by the ScholarOne Manuscripts™ system (Table 3) shows that out of all 348 manuscripts with a decision in 2018, only 25% of manuscripts accepted for publication and the rest, 75% has been rejected. This implies how stringent our control over the quality of each manuscripts.

## Figures and Tables

**Table 1:** Number of manuscript submissions by type in year 2017 and 2018

Manuscript type	2017	2018
Brief communications	29	21
Editorial	4	2
Original Article	273	290
Review Articles	41	33
Special communications	18	13

**Total**	365	359

**Table 2:** Number of manuscripts submitted, listed by country of submitting author

Country of submitting author	Manuscripts
Malaysia	142
Iran (the Islamic Republic of)	82
India	36
Indonesia	22
Nigeria	18
Saudi Arabia	10
Pakistan	9
Thailand	6
Turkey	6
Brunei Darussalam	4
Australia	2
Bangladesh	2
Egypt	2
Iraq	2
Jordan	2
Qatar	2
Syrian Arab Republic	2
Albania	1
Bosnia and Herzegovina	1
Cyprus	1
Italy	1
Libya	1
Mexico	1
Sri Lanka	1
Tunisia	1
United Arab Emirates	1
United States	1

Total	359

Source: https://mc.manuscriptcentral.com/maljms

**Table 3:** Manuscripts with a decision date between 1 Jan 2018 and 31 Dec 2018

Accepted	Accepted %	Rejected	Rejected %	Decided
87	25.0	261	75	348

Source: https://mc.manuscriptcentral.com/maljms\

## References

[1] Scimago Journal & Country Rank Journal ranking.

[2] Nour Azimah Z, Abdullah JM (2018). Malaysian Journal of Medical Sciences’ performance status in 2017 and the challenges. Malays J Med Sci.

